# ATICC: a mixed-methods study on addiction, trauma, and immigration among vulnerable young adults in the grand est youth network

**DOI:** 10.1186/s40359-025-02738-5

**Published:** 2025-05-30

**Authors:** Maha Najdini, Antoine Frigaux, Joris Mathieu, Gérard Shadili, Tamara Guenoun, Xanthie Vlachopoulou, Rose-Angélique Belot, Ariane Bazan, Florence Gressier, Marion Robin, Aziz Essadek

**Affiliations:** 1 Laboratoire INTERPSY UR4432, Université de Lorraine, 23 Boulevard Albert 1er, Nancy, 54015 France; 2 Centre Psychothérapique de Nancy, 1 Rue Dr Archambault, Laxou, 54520 France; 3 Laboratoire CRFDP UR7475, Université de Rouen Normandie, Mont-Saint-Aignan, 76821 France; 4 Department of Adolescent and Young Adult Psychiatry, Institut Mutualiste Montsouris, Paris, 75014 France; 5 Laboratoire CRPPC, Université de Lyon, Lyon, France; 6 Laboratoire PCPP, Université de Paris Cité, Paris, France; 7 Laboratoire de psychologie, université de Franche-Comté, avenue Louise-Michel, Besançon, 25000 France; 8 Moods Team, Faculté de Médecine Paris Saclay, CESP, INSERM U1018, University Paris- Saclay, Le Kremlin Bicêtre, 94275 France; 9 Hopital Saint-Maurice, Paris, France

**Keywords:** Addiction, Trauma, Immigration, Mental health, Young adults, Substance use, Foyers de jeunes travailleurs FJT

## Abstract

**Background:**

This research protocol for the study ATICC (Addiction, Trauma and Immigration, prevention and Cross-Cultural support for care with the Grand Est youth network) aims to model the complex interrelations between trauma, substance use behaviors, migration and representations of mental health among vulnerable youth residing in Transitional Housing for Young Adults. This study focuses on a specific and underexplored population, many of whom have experienced traumatic events and/or complex migratory trajectories. The ultimate objective of this research is to develop transferable models of understanding that can inform the design of prevention and support programs tailored to this demographic.

**Methods/Design:**

We adopt a tripartite methodology approach: (1) a cross-sectional study using standardized questionnaires to identify factors associated with substance use, trauma, and barriers to healthcare access; (2) an in-depth qualitative study based on semi-structured individual interviews with substance users, exploring their subjective experiences and perceptions related to substance use and mental health care; and (3) a longitudinal interventional study involving various configurations of focus groups open to residents of transitional housing structures, aiming to assess the impact of these group sessions on participants’ psychological well-being and attitudes toward care. Quantitative analyses will include descriptive and multivariate statistical tests using R, while qualitative data will be analyzed through thematic analysis with NVivo. The effectiveness of the group intervention will be evaluated using pre/post-tests, mixed models, and appropriate statistical corrections.

**Discussion:**

Analyses are expected to identify key psychosocial factors influencing addictive behaviors and mental health, highlight cultural and structural barriers to care, and assess the psychological benefits of the group intervention. The ATICC study aims to contribute to the development of culturally adapted prevention and support models for young adults in precarious situations. The results should assist healthcare professionals and policymakers in designing evidence-based interventions that effectively address the specific needs of this population.

**Trial registration:**

This study is registered with the Biomedical Research Identification Number (n°ID-RCB) assigned in France by the National Agency for the Safety of Medicines and Health Products (ANSM): 2024-A01534-43, and have received the approval from the committee for the protection of persons (CPP) Ile-de-France on November 18, 2024. Cette étude a également été déposée sur clinicaltrials (NCT06922721, date assigned April 10, 2025).

**Supplementary Information:**

The online version contains supplementary material available at 10.1186/s40359-025-02738-5.

## Background

In France, young adults facing precarious conditions can, under certain criteria, be accommodated into specific structures such as Transitional Housing Program (THP) (Foyers de Jeunes Travailleurs: FJT). These establishments provide support to individuals aged 16 to 25 who meet vulnerability criteria and have diverse life trajectories, including those transitioning from child welfare services, refugees, individuals experiencing social or familial ruptures, trainees, students, and persons with disabilities. The mission of the THP go beyond providing temporary accommodation; they actively assist young individuals in their professional integration and transition toward sustainable autonomy, as outlined by law no. 2002-2 and decree no. 2015 − 951. Therefore, they rely on a network of health, legal, and social resources to address young residents’ needs in areas such as health, budgeting, access to rights, and social life, in partnership with specialized social service actors [1].

Young adults within this demographic are particularly exposed to adverse circumstances, which can significantly impact their mental health [2]. These already challenging conditions have been further compounded by the profound and multifaceted effects of the COVID-19 pandemic, which has intensified the deterioration of mental health, particularly among young adults in precarious situations [3]. This deterioration has been notably driven by social isolation measures that disproportionately affected various groups, thereby exacerbating symptoms of anxiety and depression [4–6]. Furthermore, the pandemic has the potential to reactivate past traumas, especially among individuals with migratory backgrounds and those who have experienced challenging situations marked by familial separation [7].

The transition to adulthood, a period marked by significant psychological complexity [8], is often accompanied by various expressions of distress, including the use of psychoactive substances and engagement in addictive behaviors [9, 10]. The transition from adolescence to adulthood is characterized by the individuation process, which may reactivate issues related to past ruptures and attachment. These challenges can contribute to the development of addictive behaviors, often associated to deficits in the formation of primary bonds and difficulties in establishing interpersonal relationships [11]. From this perspective, addictive behaviors can be understood as manifestations of attachment disorders stemming from early developmental and environmental deficiencies [12]. Numerous studies have demonstrated that young adults who experienced trauma during childhood or adolescence face significantly higher risks of substance use disorders and associated health problems [13, 14]. These patterns of substance use emerge through intricate interactions among familial, social, psychological, and contextual factors [15].

For young individuals from migrant backgrounds, a substantial body of research highlights the protective effect of immigrant status [16–18]. However, this protective effect tends to diminish over time due to factors such as prolonged residence, precarious living conditions, experiences of discrimination, and acculturation-related stress [19–22]. Moreover, studies focusing on unaccompanied minors (UM)—a significant proportion of THP residents— underscore the notable vulnerability of this population to substance use. Research indicates a potential increase in substance use among individuals who had already engaged in such behaviors in their country of origin [23–25].

The described factors emphasize a critical need for mental health care among this population, raising complex issues at the intersection of social, economic, and health challenges. Due to stigma, lack of personalized support, and unfamiliarity with available resources, young residents in THP often exhibit reluctance toward seeking psychological care [26]. This hesitance appears to be particularly pronounced among youth from foreign cultural backgrounds. Extensive research has identified recurring barriers among migrant populations, including socio-economic factors such as low educational attainment, precarious living conditions, uncertain migration status, spiritual and religious beliefs, language barriers, and mistrust toward institutions perceived as remote or associated with control organizations [27, 28]. Furthermore, a systematic review [29], highlights additional obstacles and gaps in access to mental health services for refugees and asylum seekers within the European Union, such as long waiting times, insufficient availability of professional interpreters, and limited physical accessibility to services. As a result, cultural differences, stigma, and a lack of awareness of available services contribute to a significant gap between the mental health needs of this population and the care they receive [29]. Consequently, the staff at these facilities often report feeling ill-equipped to address the needs of the youth effectively, prompting a reevaluation of their practices and underscoring the necessity to enhance and expand foundational training and reexamine existing frameworks [26].

This situation underscores the need to reevaluate mental health support systems, particularly for young adults in vulnerable situations. It is within this context that our research project, ATICC (Addiction, Trauma, and Immigration: Transcultural Prevention and Support Toward Care within the Habitat Youth Network in the Grand Est Region), is situated. Conducted by researchers from the INTERPSY Laboratory (UR 4432) at the University of Lorraine, this study aims to deepen our understanding of the complex relationships between trauma, psychoactive and addictive substance use, migratory experiences, and mental health perceptions among vulnerable young adults accommodated in partner support structures and THP facilities affiliated with the Regional Union for Youth Housing (Union Régionale pour l’Habitat des Jeunes: URHAJ). The ultimate goal of this research is to develop transferable models of understanding that can inform the design of prevention and support programs. This study focuses on a specific, understudied population of vulnerable young adults, many of whom have experienced trauma and/or complex migratory trajectories. By integrating a transcultural perspective, it seeks to explore the individual and collective dynamics related to mental health and access to care, thereby addressing a critical gap in both research and practice.

In this article, we present the methodological characteristics of this research, which is built upon a solid institutional partnership framework and grounded in the contextual realities of the field, tailored to the specific needs of the target population. The study adopts a multidimensional approach, structured around a tripartite methodology that combines a quantitative cross-sectional study, a qualitative study, and an intervention-based longitudinal study. These methodologies aim to achieve several objectives: (1) To identify factors associated with addictive behaviors and their connections to various aspects of mental health and related representations. (2) To explore subjective representations and motivations underlying addictive behaviors among young people. (3) To understand the impact of migration on the life trajectories of youth and how cultural factors influence help-seeking behaviors. (4) To prevent substance use risks by demystifying mental health care representations and supporting young people in becoming autonomous in their help-seeking processes.

## Methods/design

This research project is therefore structured around three complementary phases of study (Table 1), each based on a distinct methodological approach:

- A **cross-sectional study (study 1)** that relies on a quantitative approach, conducted through the dissemination of online standardized questionnaires and scales (see: Measures) to young adults residing in partner structures at both regional and national levels. Its objective is to identify factors associated with addictive behaviors and to explore the links between trauma, migratory trajectories, food insecurity, psychological distress and perceived representations and barriers to accessing psychological care. These tools enable the rigorous collection of quantitative data and a multidimensional assessment to model the relationships between factors associated with the psychological and social challenges faced by this population. This study provides insights into general statistical trends.
- A **qualitative study (study 2)** adopting an exploratory perspective and aiming an in-depth understanding of the representations, subjective experiences, and individual and relational dynamics associated with addictive behaviors and trauma. The qualitative study builds upon the findings of Phase 1 by collecting clinical insights that provide a deeper understanding of the subjective dynamics of participants dealing with substance use issues and the obstacles encountered in their life trajectories that shape their relationship with care services.
- A **longitudinal study (study 3)** structured around a group intervention called the “Well-Being coffee group”, which operates as an open group (8 to 12 participants per session), conducted over six sessions, each lasting 1.5 to 2 h, held in the evening (6–8 PM) once a month for six months. Participation is voluntary for young residents in THP. The study aims to evaluate the impact of this group discussion format on participants’ psychological well-being, their perceptions of mental health, and their access to care.

Participants are assessed at multiple time points (T0: prior to the program, T1: mid-program, T2: end of the program, T3: post-program follow-up). These longitudinal evaluations, based on standardized tools, will enable a detailed analysis of participants’ well-being progression and the specific effects attributable to the group intervention. This study is part of an action-research framework, integrating clinical intervention with the production of scientific knowledge while offering transferable recommendations for other support programs aimed at vulnerable young adults.

**Table 1:** Summary table of the research methodology

	Study 1	Study 2	Study 3
Type of study	Quantitative	Qualitative	Longitudinale
Method	Cohort study using self-administered questionnaires	Semi-structured interviews Rorschach test Adult Attachment interview (AAI)	Group intervention Self-administrated questionnaires
Eligibility criteria	Participant aged > 18 Resident in a Housing center Fluent in French adult participants who are not under guardianship (tutelle) or curatorship (curatelle).	Participant aged > 18 Fluent in French Any substance user Migratory background	Same as study 1
Exclusion criteria	Minors Do not speak and read French Under Guardianship or Curatorship	Minors Do not speak and read French Under Guardianship or Curatorship	Minors Do not speak and read French Under Guardianship or Curatorship
Main Outcomes	Substance use prevalence Mental health representations Mental health aspects (anxiety, depression) Childhood trauma	Substance use, trauma and migration trajectory (semi-structure interview) Attachment style (AAI tool) Psychological functioning (Rorschach test)	Substance use Perception of mental health care Wellness
Analysis	Quantitative analysis	Thematic analysis	Quantitative and qualitative analysis

### Participants

#### Study 1

We based our calculations on an approximate population size of 60,000 young individuals supported throughout the previous year (Year N-1) across the entire territory. The margin of error was set at 5%, with a confidence level of 95%. We applied Cochran’s formula (Z² × p × (1-p) / E²) to determine the required sample size. Accordingly, the sample size necessary for this cross-sectional study is 382 respondents.

#### Study 2

We will base our qualitative study on a sample of 30 young volunteers recruited independently of the cross-sectional study, following the procedures outlined below. However, participant inclusion will continue until data saturation is reached. Data saturation will be considered achieved when the main themes emerge, meaning that all relevant themes necessary to address the research questions have been identified in the collected data.

#### Study 3

For this study, we estimate a strong effect size of 0.7 (Cohen’s d) with a significance level of 0.05. Based on these parameters, and using the software G*Power 3.1, we determined that 55 participants would be required for each group modality, resulting in a total of 165 participants. To account for an estimated 10% dropout rate, we will include 182 young individuals in the study.

### Procedures

#### Study 1

A national deployment will be conducted in collaboration with all partners and youth centers. Recruitment will be facilitated via email distribution. Young residents of Youth transition Centers across the country will receive an email from the National Union for Youth Housing (Union Nationale pour l’Habitat des Jeunes: UNHAJ). This email will include detailed information about the research and a link to access the study. Upon accessing the study’s homepage, participants will encounter an introductory note outlining the study, its objectives, a contact point for further information, and a link to open the questionnaire. Clicking the link will direct them to a consent form with a binary choice: “Yes” or “No,” responding to the question: “Do you consent to participate in this research?“. Participants who select “Yes” will activate the questionnaire and proceed with the study, while those who select “No” will not be able to participate.

#### Study 2

The qualitative study will focus on a sample of young residents from all the structures involved in this research. Inclusion criteria require participants to be adults, French-speaking, and affected by substance use issues. Participants who consent to take part in the study, after reviewing the information notice provided at least 15 days prior to the first interview to allow for reflection, will meet with one of the investigators from the research team. The investigator will present the consent form, detailing the study’s objectives, methodology, duration, potential constraints, and foreseeable risks, while addressing any questions from the participant.

Each participant will undergo three sessions of approximately one hour each: a semi-structured interview, administration of the Rorschach test, and the Adult Attachment Interview (AAI). All interviews will be conducted by the same investigator who conducted the initial session to foster a trusting environment. The study in each structure will be conducted in a secure and confidential setting for participants. All three sessions will be audio-recorded using a voice recorder, transcribed, anonymized, and subsequently destroyed to ensure confidentiality.

#### Study 3

The longitudinal study will follow the same inclusion criteria as the qualitative study (Study 2). However, participation in the group sessions is open to all volunteers for each session, regardless of whether they are substance users. Informed consent will be systematically obtained before each session, ensuring fully informed participation. Promotion of the groups will be conducted through widespread dissemination of informational posters, email communications via the management of the partner structures, and during informational meetings. A follow-up meeting will be organized 15 days later to conduct the initial T0 evaluation with the volunteers.

**Fig. 1:**
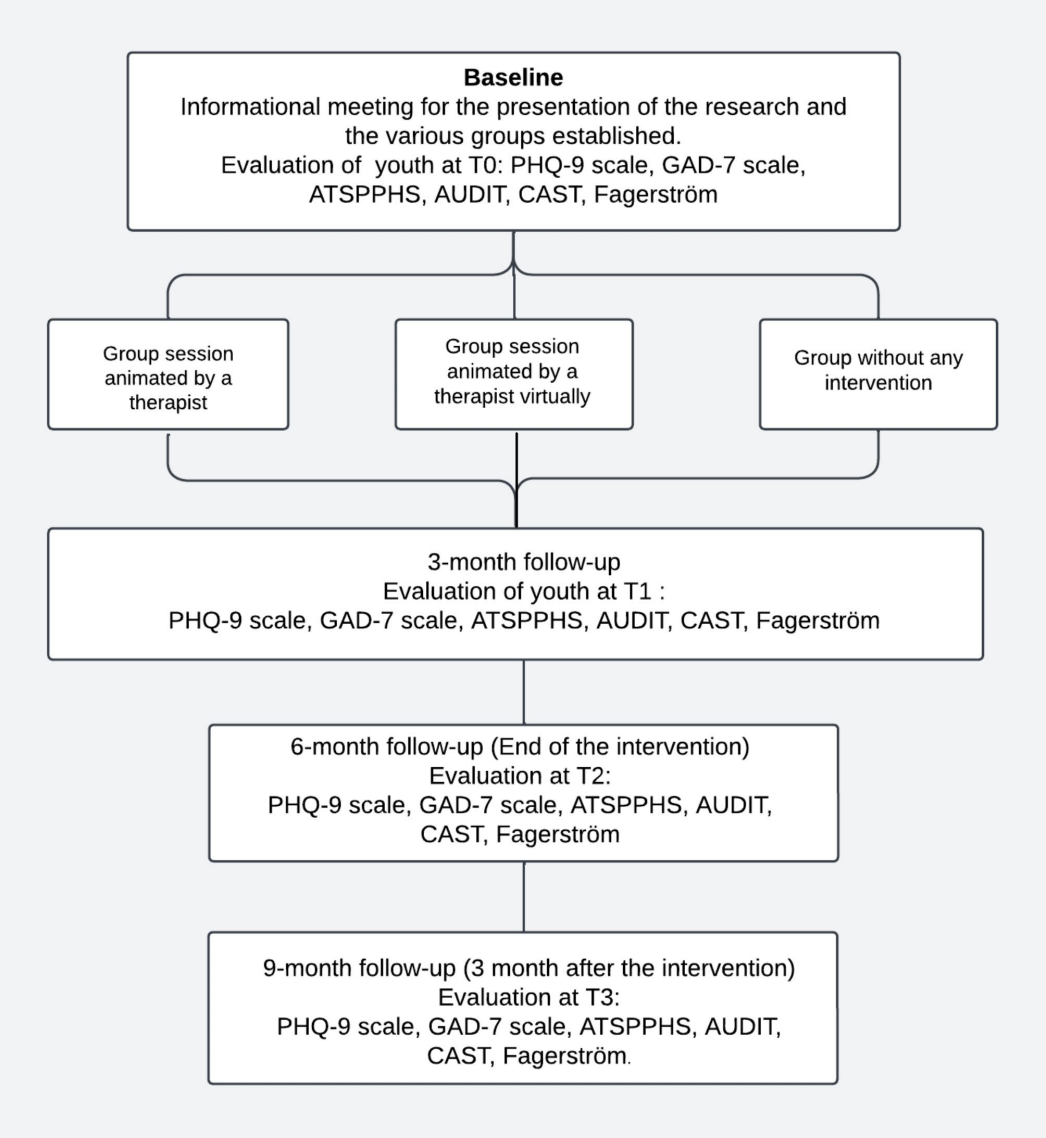
Procedure flowchart for longitudinal study (study 3)

The “Well-Being coffee group” intervention is implemented in several formats (see Fig. 1). Some groups will take place in person, others via videoconference, all conducted by the same psychologist throughout the sessions. Additionally, a control group will consist of participants who underwent the initial evaluation (T0) but did not attend any sessions. The inclusion of this control group allows for the assessment of changes in psychological and behavioral outcomes over time, comparing those who received the intervention to those who did not. The choice of this comparator was made based on feasibility and ethical considerations, ensuring that participants were not deprived of potential care while maintaining an adequate framework for evaluating the intervention’s effects. Across all formats, participation is voluntary, following a prior information phase and the signing of an informed consent form. Participants will complete standardized evaluation questionnaires at multiple time points (T0, T1, T2, T3).

The discussion groups aim to provide a supportive and open space for free expression. Their objective is to allow participants to articulate their thoughts, emotions, and personal experiences, as well as those evoked by group discussions on various themes, including identity, substance use, migratory experiences, and mental health. The impact of the intervention will be assessed regularly using standardized tools to measure levels of anxiety, depression, and changes in attitudes and willingness to seek help. Finally, a global satisfaction scale will be used at the end of the sessions to collect participants’ subjective feedback on their experiences within the groups (see Fig. 1).

### Measures

#### Study 1

The questionnaire will consist of two sections: one addressing sociodemographic questions and another utilizing standardized scales. The scales included are as follows:

**Food Insecurity Experience Scale (FIES)** [30–32]: An 8-item measure assessing the severity of food insecurity experienced, based on difficulties accessing adequate food.

**Childhood Trauma Questionnaire Short Form (CTQ-SF)** [33, 34]: A 28-item instrument evaluating various types of childhood trauma, including emotional, physical, sexual, and psychological trauma. An additional item has been included in this scale.

**Hopkins Symptom Check List 25 (HSCL 25)** [35–37]: A 25-item symptom inventory measuring anxiety (10 items) and depression (15 items).

**Barriers to Access to Care Evaluation (BACE-3)** [38, 39]: A 30-item tool assessing barriers to accessing mental health care, including stigma-related obstacles.

**Nightmare Severity Index (NSI)** [40]: A 9-item scale evaluating the nature of insomnia, satisfaction with sleep, and the impact of sleep disturbances on daily life.

**Alcohol Use Disorders Identification Test (AUDIT)** [41, 42]: A 10-item questionnaire designed to identify individuals at risk of alcohol addiction, addressing alcohol consumption, dependence, and related problems.

**Fagerström Test for Nicotine Dependence** [43, 44]: A 6-item questionnaire assessing the level of nicotine dependence in smokers.

**Cannabis Abuse Screening Test (CAST)** [45]: A 6-item screening tool to identify problematic cannabis use, particularly among adolescents and young adults, focusing on consumption habits and related issues over the past 12 months.

**Geometric Categorization Task (GeoCAT)** [46, 47]: A 6-item tool measuring primary and secondary cognitive processes.

#### Study 2

A semi-structured interview guide was developed based on a review of existing literature and preliminary interviews. The questions focus on participants’ life narratives and their experiences with substance use. The administration of the Rorschach test and the Adult Attachment Interview (AAI) will also be conducted. The Rorschach test is a “projective” psychological assessment composed of 10 cards depicting black-and-white and color inkblots. It allows for the assessment of an individual’s psychological functioning concerning certain fundamental competencies expressed through their engagement with the test material, such as their perception style, emotional expression, approach to interpersonal relationships, and significant individual characteristics and issues that reflect a singular mode of psychological organization [48]. The Adult Attachment Interview (AAI) is a 20-question interview designed to assess an individual’s attachment representations based on their recollections of early caregiving experiences. The responses are transcribed and analyzed using a rigorous coding system, classifying individuals into attachment categories such as secure, dismissing, preoccupied, or unresolved regarding trauma or loss [49, 50].

#### Study 3

**Generalized Anxiety Disorder (GAD-7)** [51–53]: A 7-item self-report questionnaire designed to assess the patient’s anxiety levels over the previous two weeks.

**Patient Health Questionnaire-9 (PHQ-9)** [54, 55]: A 9-item tool used for depression screening that also provides an indication of the severity of depressive symptoms.

**Alcohol Use Disorders Identification Test (AUDIT)** [41, 42]: A 10-item questionnaire designed to determine whether an individual is at risk of alcohol addiction. The items assess alcohol consumption, dependency, and related problems.

**Fagerström Test for Nicotine Dependence** [43, 44]: A 6-item questionnaire created to evaluate the level of nicotine dependence among smokers.

**Cannabis Abuse Screening Test (CAST)** [45]: A screening tool designed to identify problematic cannabis use, particularly among adolescents and young adults. It consists of six questions addressing consumption habits and associated problems over the past 12 months.

**Attitudes Toward Seeking Professional Psychological Help Short Form (ATSPPH-S)** [56, 57]: A 10- items psychometric scale designed to measure individuals’ attitudes toward seeking professional psychological help.

### Data analysis

#### Study 1

The research team will conduct statistical analyses using R. The cross-sectional study will characterize the study population through descriptive and multivariate analyses (e.g., Chi-square/Fisher’s exact tests, correlation tests, ANOVA, etc.). We will adhere to the STROBE guidelines for epidemiological studies [58]. A significance level of 0.05 will be applied for statistical analysis.

#### Study 2

A thematic analysis of the data from the semi-structured interviews will be conducted using NVivo 15 software. Moreover, the findings from the Rorschach test will be analyzed using a methodology for interpreting projective material [48], with the aim of highlighting underlying psychic processes, particularly those related to attachment dynamics early traumas, the defensive mechanisms of participants. Additionally, the interviews reported from the AAI assessment will be coded by MN and AF under the supervision of a certified reliable coder R-AB using the Adult Attachment Scoring and Classification System [49].

#### Study 3

For the group intervention, a Bonferroni correction will be applied to all *p*-values in t-tests. Cronbach’s alpha will be used to assess the reliability of the scales. Various analyses will be conducted to evaluate pre- and post-intervention effects as well as dose-response effects, including Student’s t-tests, repeated-measures ANOVA, and linear regression.

Primary and secondary outcomes will be analyzed using linear mixed models, incorporating a random group effect if estimation permits. The primary outcome measure is the variation in the total score on the ATSPPH-S at 9 months, calculated using dimensional scoring algorithms. Changes in the primary outcome and the relative effectiveness of the four intervention modalities will be assessed using mixed regression models, utilizing all available data while accounting for participant, time, and group levels. The underlying distribution for the mixed regression model will be determined based on residual distribution.

Per-protocol analyses will also be performed to test robustness. ANCOVA analyses may be conducted as needed to control for confounding variables that could influence the results. If data distribution is non-parametric, analyses such as Mann-Whitney, Wilcoxon, and Kruskal-Wallis tests will be employed.

Missing data will be reported and managed using appropriate imputation methods depending on their proportion and classification (MCAR, MAR or MNAR). However, if the proportion of missing data is very large (> 40%), we will present only the results of the complete case analysis [59].

### The roles of stakeholders of the research

The investigation team is comprised of university researchers and clinical psychologists who have experience in working with young people AE, AF & MN. The structures involved in this research project will also contribute by facilitating the dissemination of the online cross-sectional study at both the regional and national levels. The social worker teams within each structure will help circulate information about the various phases of the study, invite the young people they support to participate, and assist in organizing initial meetings with participants. Additionally, JM, FG, GS and MR will support the data analysis process, with R-AB overseeing the analysis of data collected through the AAI, AF and XV supervising the analysis of Rorschach test data. The scientific director of this research AE takes overall responsibility for the project including oversight of the budget and ensuring that ethical processes are followed.

### Ethical considerations

We obtained approval from the Committee for the Protection of Persons (CPP) Ile de France VI in November 2024 to conduct this study, classified as a Minimal Risk Interventional Research (RIPH 2) study, and registered under number ID-RCB: 2024-A01534-43.

The ATICC research project is also registered in the data processing registry of the University of Lorraine under number 2024 − 358 of 25/07/2024, and adheres to the Reference Methodology MR-001, a regulatory framework defined by the French Data Protection Authority or National Commission on Informatics and Liberty (CNIL) for research involving personal data in the health domain. These registrations ensure that the processing of participants’ personal data complies with ethical and legal standards for data protection, in accordance with the General Data Protection Regulation (RGPD) and the internal guidelines of the University of Lorraine. Any identifying information will be removed before data analysis to maintain anonymity.

### Data monitoring and management

No data monitoring committee has been appointed, as data monitoring responsibilities are shared among the research team members. Data entry will be verified twice to ensure accuracy, and data quality will be assured through range checks.

### Safety considerations

No adverse effects are expected. The questionnaires selected for this study have widely been used in previous research with self-administrated protocols. Meanwhile, the semi-structures interviews from study 2 and focus groups from study 3 may bring up difficult life events. In cases where participation leads to distress or emotional discomfort, participants will receive support and compassionate guidance from the investigator/psychologist conducting the interview, as well as during an individual debriefing following the group sessions. If an urgent need for care is identified, participants will be referred to appropriate socio-medical services for further support.

The findings of the study will be published and shared with the scientific community including scientific publications, conference posters and oral presentations in national and international conferences.

Withdrawal from any phase of the study will be considered if participants revoke their consent during data collection, if they discontinue attendance at research interviews, or if they cease participation in group sessions. Withdrawal is entirely voluntary and will not result in any consequences, ensuring adherence to ethical research principles.

## Discussion

The objective of this study is to deepen the understanding of substance use behaviors among young individuals and the barriers to accessing healthcare, with the aim of improving and adapting support mechanisms in Youth Transition Centers. The anticipated results will contribute to the modeling of the relationships between trauma, migration, and addiction, while broadly informing treatment and support practices within these structures to ensure they are both accessible and acceptable.

In this research, we expect to find a significant prevalence of traumatic histories, psychological distress symptoms, and barriers to accessing mental health care among young substance users. We hypothesize that substance use may serve as an adaptive response to disruptions in familial and cultural contexts, shedding light on attachment-related issues. Previous studies have demonstrated the impact of traumatic experiences on the development and persistence of behaviors associated with psychoactive substance use. These findings also highlight the need to move beyond traditional abstinence-focused approaches, which are often insufficient to address the underlying causes of addictive behaviors [60–62]. Other studies suggest the self-medication hypothesis, where substance use alleviates trauma-related symptoms, thereby reinforcing dependency and reducing the likelihood of seeking help [63, 64]. A trauma-centered approach, therefore, emerges as essential for providing comprehensive and better-tailored care to meet the needs of young individuals [61].

We suggest that cultural factors significantly influence psychoactive substance use behaviors among young people as well as their perceptions of mental health care. Research indicates that cultural factors are associated with variations in substance use patterns [65, 66]. Regarding healthcare, a systematic review on the perception of mental health interventions among adolescents and young adults revealed that their beliefs about care and access to care are often stigmatized, misinformed, and partially shaped by cultural factors, with limited understanding of the origins of these perceptions [67]. Studies emphasize the importance of viewing cultural diversity not as a barrier but as an opportunity to enhance mental health care through ethnocultural approaches that integrate traditional knowledge and community resources [68].

Furthermore, the central aspect of this research lies in integrating the cultural dimension into understanding issues related to substance use and implementing group-based interventions. Numerous studies have underscored the importance of cultural adaptation in general and specifically in the treatment of substance use disorders [69–71]. However, this approach remains limited if it is not accompanied by efforts to raise young people’s awareness of the importance of mental health care.

We therefore expect an improvement in the well-being of young participants engaged in group-based interventions and a positive shift in their perceptions of psychological care, potentially leading to an increased demand for such services. Additionally, virtual group settings are expected to enhance accessibility and strengthen the effectiveness of interventions.

Ultimately, this research project aspires to develop comprehensive, evidence-based intervention and prevention strategies, aimed at more effectively addressing the specific needs of young residents in transitional youth centers.

### Trial status

The present protocol corresponds to version 2024-A01534-43, validated on November 28, 2024, by the Committee for the Protection of Persons Ile de France VI. Recruitment for the ATICC project is ongoing since January 2025 and is expected to be complete in December 2025. This timeline reflects the structured phases of participant enrollment across the three studies, ensuring the project adheres to its methodological and ethical framework as approved by the regulatory authorities. Any substantial modification to the protocol will require prior approval from the CPP before implementation. All amendments will be documented, justified, and communicated to relevant stakeholders.

## Electronic supplementary material

Below is the link to the electronic supplementary material.

Supplementary Material 1

## Data Availability

The data collected as part of the ATICC study will be anonymized and secured, with access strictly limited to authorized members of the research team, identified in accordance with GDPR-compliant procedures and the reference methodology MR-001 (Commission nationale de l’informatique et des libertés). These data will be stored on secure servers at the University of Lorraine, with access restrictions defined to ensure their confidentiality. Data will be made accessible to interested parties upon reasonable request submitted to the corresponding author.

## Abbreviations

ATICC: Addiction, Trauma, Immigration, and Cross-Cultural support for care
URHAJ: Union Régionale pour l’Habitat des Jeunes
UNHAJ: Union Nationale pour l’Habitat des Jeunes
THP: Foyers de Jeunes Travailleurs
AAI: Adult Attachment Interview
STROBE: Strengthening the Reporting of Observational Studies in Epidemiology
FIES: Food Insecurity Experience Scale
CTQ-SF: Childhood Trauma Questionnaire Short Form
HSCL-25: Hopkins Symptom Check List 25
BACE-3: Barriers to Access to Care Evaluation (Version 3)
NSI: Nightmare Severity Index
AUDIT: Alcohol Use Disorders Identification Test
CAST: Cannabis Abuse Screening Test
GAD-7: Generalized Anxiety Disorder 7-item scale
PHQ-9: Patient Health Questionnaire-9
ATSPPH-S: Attitudes Toward Seeking Professional Psychological Help Short Form
ANOVA: Analysis of Variance
