# Supplementary material for: ATICC: a mixed-methods study on addiction, trauma, and immigration among vulnerable young adults in the grand est youth network
Source: BMC Psychol. 2025 May 30;13:582. doi: 10.1186/s40359-025-02738-5 (PMC12125730; doi:10.1186/s40359-025-02738-5)
Supplement: Supplementary file 1 — Supplementary Material 1 [file 40359_2025_2738_MOESM1_ESM.docx]

# Additional files

**SPIRIT Guidance**

|  |  | **Reporting Item** | **Page and Line Number** | **Reason if not applicable** |
| --- | --- | --- | --- | --- |
| **Administrative information** | | | | |
| Title | [#1](https://www.goodreports.org/reporting-checklists/spirit/info/#1) | Descriptive title identifying the study design, population, interventions, and, if applicable, trial acronym | l2, p1 |  |
| Trial registration | [#2a](https://www.goodreports.org/reporting-checklists/spirit/info/#2a) | Trial identifier and registry name. If not yet registered, name of intended registry | l65, p2 |  |
| Trial registration: data set | [#2b](https://www.goodreports.org/reporting-checklists/spirit/info/#2b) | All items from the World Health Organization Trial Registration Data Set |  | This study has been registered in an official trial registry (Biomedical Research Identification Number (n°ID-RCB) assigned in France by the National Agency for the Safety of Medicines and Health Products (ANSM): 2024-A01534-43).  All WHO Trial Registration Data Set items are covered in the study protocol, including study objectives, eligibility criteria, intervention details, and data collection methods. |
| Protocol version | [#3](https://www.goodreports.org/reporting-checklists/spirit/info/#3) | Date and version identifier |  |  |
| Funding | [#4](https://www.goodreports.org/reporting-checklists/spirit/info/#4) | Sources and types of financial, material, and other support | l497, p16 |  |
| Roles and responsibilities: contributorship | [#5a](https://www.goodreports.org/reporting-checklists/spirit/info/#5a) | Names, affiliations, and roles of protocol contributors | L502 p16 |  |
| Roles and responsibilities: sponsor contact information | [#5b](https://www.goodreports.org/reporting-checklists/spirit/info/#5b) | Name and contact information for the trial sponsor | L27 p1 |  |
| Roles and responsibilities: sponsor and funder | [#5c](https://www.goodreports.org/reporting-checklists/spirit/info/#5c) | Role of study sponsor and funders, if any, in study design; collection, management, analysis, and interpretation of data; writing of the report; and the decision to submit the report for publication, including whether they will have ultimate authority over any of these activities | L376-378 p12  l495 p16 |  |
| Roles and responsibilities: committees | [#5d](https://www.goodreports.org/reporting-checklists/spirit/info/#5d) | Composition, roles, and responsibilities of the coordinating centre, steering committee, endpoint adjudication committee, data management team, and other individuals or groups overseeing the trial, if applicable (see Item 21a for data monitoring committee) | N/A | l422, p14 “No data monitoring committee has been appointed, as data monitoring responsibilities are shared among the research team members”.  The ATICC study does not have a formal external oversight committee. However, scientific and methodological supervision is ensured internally by the research team.  The principal investigator (AE) oversees the overall supervision of the project, monitors funding, and validates methodological decisions.  The co-investigators (MN, AF) are responsible for organizing the different phases of the study and managing the evaluation protocols.  Statistical analyses are overseen by AE, MN and FG, ensuring compliance with methodological standards.  Data from the Adult Attachment Interview (AAI) are supervised by RB, an expert in this tool. |
| **Introduction** |  |  |  |  |
| Background and rationale | [#6a](https://www.goodreports.org/reporting-checklists/spirit/info/#6a) | Description of research question and justification for undertaking the trial, including summary of relevant studies (published and unpublished) examining benefits and harms for each intervention | Background  l74, p3 |  |
| Background and rationale: choice of comparators | [#6b](https://www.goodreports.org/reporting-checklists/spirit/info/#6b) | Explanation for choice of comparators | l257, p8 |  |
| Objectives | [#7](https://www.goodreports.org/reporting-checklists/spirit/info/#7) | Specific objectives or hypotheses | l155, p5 |  |
| Trial design | [#8](https://www.goodreports.org/reporting-checklists/spirit/info/#8) | Description of trial design including type of trial (eg, parallel group, crossover, factorial, single group), allocation ratio, and framework (eg, superiority, equivalence, non-inferiority, exploratory) | l162-195, p5-6 | The trial follows an exploratory framework, aiming to generate preliminary insights into mental health care among young adults. |
| **Methods: Participants, interventions, and outcomes** | | | | |
| Study setting | [#9](https://www.goodreports.org/reporting-checklists/spirit/info/#9) | Description of study settings (eg, community clinic, academic hospital) and list of countries where data will be collected. Reference to where list of study sites can be obtained | L142 p4, l167 p5, l231 P7 |  |
| Eligibility criteria | [#10](https://www.goodreports.org/reporting-checklists/spirit/info/#10) | Inclusion and exclusion criteria for participants. If applicable, eligibility criteria for study centres and individuals who will perform the interventions (eg, surgeons, psychotherapists) | Table 1 p6 |  |
| Interventions: description | [#11a](https://www.goodreports.org/reporting-checklists/spirit/info/#11a) | Interventions for each group with sufficient detail to allow replication, including how and when they will be administered | L217-272, p7-8 |  |
| Interventions: modifications | [#11b](https://www.goodreports.org/reporting-checklists/spirit/info/#11b) | Criteria for discontinuing or modifying allocated interventions for a given trial participant (eg, drug dose change in response to harms, participant request, or improving / worsening disease) | L396-404, p13  l408-411, p13 |  |
| Interventions: adherance | [#11c](https://www.goodreports.org/reporting-checklists/spirit/info/#11c) | Strategies to improve adherence to intervention protocols, and any procedures for monitoring adherence (eg, drug tablet return; laboratory tests) | l219-222, 232-237, l249-252 p7-8  l370-374, p12 |  |
| Interventions: concomitant care | [#11d](https://www.goodreports.org/reporting-checklists/spirit/info/#11d) | Relevant concomitant care and interventions that are permitted or prohibited during the trial | N/A | There are no restrictions on concomitant care or interventions during the study and no specific interventions are prohibited. |
| Outcomes | [#12](https://www.goodreports.org/reporting-checklists/spirit/info/#12) | Primary, secondary, and other outcomes, including the specific measurement variable (eg, systolic blood pressure), analysis metric (eg, change from baseline, final value, time to event), method of aggregation (eg, median, proportion), and time point for each outcome. Explanation of the clinical relevance of chosen efficacy and harm outcomes is strongly recommended | Table 1 p6 – "Main Outcomes"  l276-331, p10-11 – "Measures"  l332-366, p11-P12 – "Data analysis – Primary and secondary outcomes"  l421-426 p13  l447-451 p14 |  |
| Participant timeline | [#13](https://www.goodreports.org/reporting-checklists/spirit/info/#13) | Time schedule of enrolment, interventions (including any run-ins and washouts), assessments, and visits for participants. A schematic diagram is highly recommended (see Figure) | l218-226, p7  l232-240 p7  l252-264, p10  l273, p9 – "Figure 1: Procedure flowchart for longitudinal study" |  |
| Sample size | [#14](https://www.goodreports.org/reporting-checklists/spirit/info/#14) | Estimated number of participants needed to achieve study objectives and how it was determined, including clinical and statistical assumptions supporting any sample size calculations | L197-215, p6-7 |  |
| Recruitment | [#15](https://www.goodreports.org/reporting-checklists/spirit/info/#15) | Strategies for achieving adequate participant enrolment to reach target sample size | l218-228, p7 – "Recruitment procedures"  l249-252, p8 – "Recruitment for Study 3" |  |
| **Methods: Assignment of interventions (for controlled trials)** | | | | |
| Allocation: sequence generation | [#16a](https://www.goodreports.org/reporting-checklists/spirit/info/#16a) | Method of generating the allocation sequence (eg, computer-generated random numbers), and list of any factors for stratification. To reduce predictability of a random sequence, details of any planned restriction (eg, blocking) should be provided in a separate document that is unavailable to those who enrol participants or assign interventions | N/A | This study does not rely on randomized allocation.  Participants in the focus groups (Study 3) are recruited on a voluntary basis and can choose between an in-person or video conferencing format.  A natural control group consists of participants who complete the initial assessment but do not attend the focus groups.  No randomized allocation sequence is generated. |
| Allocation concealment mechanism | [#16b](https://www.goodreports.org/reporting-checklists/spirit/info/#16b) | Mechanism of implementing the allocation sequence (eg, central telephone; sequentially numbered, opaque, sealed envelopes), describing any steps to conceal the sequence until interventions are assigned | N/A | Allocation concealment is not applicable to this study, as participants voluntarily select their intervention groups rather than being randomly assigned |
| Allocation: implementation | [#16c](https://www.goodreports.org/reporting-checklists/spirit/info/#16c) | Who will generate the allocation sequence, who will enrol participants, and who will assign participants to interventions | N/A | There is no need to implement allocation, as group assignment is based on participant choice |
| Blinding (masking) | [#17a](https://www.goodreports.org/reporting-checklists/spirit/info/#17a) | Who will be blinded after assignment to interventions (eg, trial participants, care providers, outcome assessors, data analysts), and how | N/A | Blinding is not applicable in this study, as participants are aware of their group assignment (voluntary participation in focus groups).  The facilitators and researchers conducting the intervention are also aware of group allocation.  However, outcome assessors and data analysts may be blinded to the group allocation during analysis to minimize bias. |
| Blinding (masking): emergency unblinding | [#17b](https://www.goodreports.org/reporting-checklists/spirit/info/#17b) | If blinded, circumstances under which unblinding is permissible, and procedure for revealing a participant’s allocated intervention during the trial | N/A | Unblinding procedures are not applicable in this study, as no blinding is implemented.  Participants, facilitators, and researchers are aware of group assignments.  If data analysts are blinded to group allocation, this will remain unchanged throughout the analysis process. |
| **Methods: Data collection, management, and analysis** | | | | |
| Data collection plan | [#18a](https://www.goodreports.org/reporting-checklists/spirit/info/#18a) | Plans for assessment and collection of outcome, baseline, and other trial data, including any related processes to promote data quality (eg, duplicate measurements, training of assessors) and a description of study instruments (eg, questionnaires, laboratory tests) along with their reliability and validity, if known. Reference to where data collection forms can be found, if not in the protocol | L276-331, p9-11 – "Measures"  l332-366, p11-p12 – "Data Analysis"  L368-369 p12, l374-376 p12 |  |
| Data collection plan: retention | [#18b](https://www.goodreports.org/reporting-checklists/spirit/info/#18b) | Plans to promote participant retention and complete follow-up, including list of any outcome data to be collected for participants who discontinue or deviate from intervention protocols | L408-411 p13 |  |
| Data management | [#19](https://www.goodreports.org/reporting-checklists/spirit/info/#19) | Plans for data entry, coding, security, and storage, including any related processes to promote data quality (eg, double data entry; range checks for data values). Reference to where details of data management procedures can be found, if not in the protocol | L379-391, p12 – "Ethical Considerations"  L393-395, p13 – "Data Monitoring and Management" |  |
| Statistics: outcomes | [#20a](https://www.goodreports.org/reporting-checklists/spirit/info/#20a) | Statistical methods for analysing primary and secondary outcomes. Reference to where other details of the statistical analysis plan can be found, if not in the protocol | l348-358, p11-12 – "Data Analysis" |  |
| Statistics: additional analyses | [#20b](https://www.goodreports.org/reporting-checklists/spirit/info/#20b) | Methods for any additional analyses (eg, subgroup and adjusted analyses) | L359-362, p12– "Data Analysis" |  |
| Statistics: analysis population and missing data | [#20c](https://www.goodreports.org/reporting-checklists/spirit/info/#20c) | Definition of analysis population relating to protocol non-adherence (eg, as randomised analysis), and any statistical methods to handle missing data (eg, multiple imputation) | L363-366 p12 – "Data Analysis" |  |
| **Methods: Monitoring** | | | | |
| Data monitoring: formal committee | [#21a](https://www.goodreports.org/reporting-checklists/spirit/info/#21a) | Composition of data monitoring committee (DMC); summary of its role and reporting structure; statement of whether it is independent from the sponsor and competing interests; and reference to where further details about its charter can be found, if not in the protocol. Alternatively, an explanation of why a DMC is not needed | N/A  L393-395, p13 | No formal Data Monitoring Committee (DMC) has been appointed, as data monitoring responsibilities are shared among the research team members.  Data entry will be verified twice to ensure accuracy, and data quality will be assured through range checks. |
| Data monitoring: interim analysis | [#21b](https://www.goodreports.org/reporting-checklists/spirit/info/#21b) | Description of any interim analyses and stopping guidelines, including who will have access to these interim results and make the final decision to terminate the trial | N/A | No interim analysis or stopping guidelines are planned for this study, as it does not involve high-risk interventions.  The study will proceed as planned unless significant unforeseen circumstances require modifications. |
| Harms | [#22](https://www.goodreports.org/reporting-checklists/spirit/info/#22) | Plans for collecting, assessing, reporting, and managing solicited and spontaneously reported adverse events and other unintended effects of trial interventions or trial conduct | L397-404, p13 – "Safety Considerations" |  |
| Auditing | [#23](https://www.goodreports.org/reporting-checklists/spirit/info/#23) | Frequency and procedures for auditing trial conduct, if any, and whether the process will be independent from investigators and the sponsor | N/A | The study will be subject to internal monitoring by the University of Lorraine, ensuring compliance with ethical and methodological guidelines.  Periodic reviews will be conducted by the principal investigator and research team to verify adherence to protocols and data integrity. |
| **Ethics and dissemination** | | | | |
| Research ethics approval | [#24](https://www.goodreports.org/reporting-checklists/spirit/info/#24) | Plans for seeking research ethics committee / institutional review board (REC / IRB) approval | L380-391, p12 |  |
| Protocol amendments | [#25](https://www.goodreports.org/reporting-checklists/spirit/info/#25) | Plans for communicating important protocol modifications (eg, changes to eligibility criteria, outcomes, analyses) to relevant parties (eg, investigators, REC / IRBs, trial participants, trial registries, journals, regulators) | L461-463, p14 |  |
| Consent or assent | [#26a](https://www.goodreports.org/reporting-checklists/spirit/info/#26a) | Who will obtain informed consent or assent from potential trial participants or authorised surrogates, and how (see Item 32) | l235-237 p7, l248-249 p 8 |  |
| Consent or assent: ancillary studies | [#26b](https://www.goodreports.org/reporting-checklists/spirit/info/#26b) | Additional consent provisions for collection and use of participant data and biological specimens in ancillary studies, if applicable | N/A | No ancillary studies involving additional data collection or biological specimens are planned in this research. Therefore, no additional consent provisions are required. |
| Confidentiality | [#27](https://www.goodreports.org/reporting-checklists/spirit/info/#27) | How personal information about potential and enrolled participants will be collected, shared, and maintained in order to protect confidentiality before, during, and after the trial | L240-244 p8  L488-494, p16 |  |
| Declaration of interests | [#28](https://www.goodreports.org/reporting-checklists/spirit/info/#28) | Financial and other competing interests for principal investigators for the overall trial and each study site | L496, p16 |  |
| Data access | [#29](https://www.goodreports.org/reporting-checklists/spirit/info/#29) | Statement of who will have access to the final trial dataset, and disclosure of contractual agreements that limit such access for investigators | L488-489, p16 |  |
| Ancillary and post trial care | [#30](https://www.goodreports.org/reporting-checklists/spirit/info/#30) | Provisions, if any, for ancillary and post-trial care, and for compensation to those who suffer harm from trial participation | N/A | No specific post-trial care is planned as part of this study. However, participants who require additional support will be referred to appropriate mental health and social care services. |
| Dissemination policy: trial results | [#31a](https://www.goodreports.org/reporting-checklists/spirit/info/#31a) | Plans for investigators and sponsor to communicate trial results to participants, healthcare professionals, the public, and other relevant groups (eg, via publication, reporting in results databases, or other data sharing arrangements), including any publication restrictions | l405-407, p13 |  |
| Dissemination policy: authorship | [#31b](https://www.goodreports.org/reporting-checklists/spirit/info/#31b) | Authorship eligibility guidelines and any intended use of professional writers | l502-507, p16 |  |
| Dissemination policy: reproducible research | [#31c](https://www.goodreports.org/reporting-checklists/spirit/info/#31c) | Plans, if any, for granting public access to the full protocol, participant-level dataset, and statistical code | L493-494, p16 |  |
| **Appendices** | | | | |
| Informed consent materials | [#32](https://www.goodreports.org/reporting-checklists/spirit/info/#32) | Model consent form and other related documentation given to participants and authorised surrogates | Appendix 1 p 30-32  Appendix 2 p 33-35  Appendix 3 p36-37 |  |
| Biological specimens | [#33](https://www.goodreports.org/reporting-checklists/spirit/info/#33) | Plans for collection, laboratory evaluation, and storage of biological specimens for genetic or molecular analysis in the current trial and for future use in ancillary studies, if applicable | N/A | No biological specimens will be collected, analyzed, or stored as part of this study. Therefore, this item is not applicable. |

It is strongly recommended that this checklist be read in conjunction with the SPIRIT 2013 Explanation & Elaboration for important clarification on the items. Amendments to the protocol should be tracked and dated. The SPIRIT checklist is copyrighted by the SPIRIT Group under the Creative Commons “[Attribution-NonCommercial-NoDerivs 3.0 Unported](http://www.creativecommons.org/licenses/by-nc-nd/3.0/)” license. This checklist can be completed online using https://www.goodreports.org/, a tool made by the EQUATOR Network in collaboration with Penelope.ai.

**Appendix 1**

**Information notice (qualitative study)**

**Project ATICC**

**N° ID-RCB : 2024-A01534-43**

Please read this notice carefully and ask any questions you may have. You can then decide whether or not you want to participate in this research.

**Title of the Study:** Addiction, Trauma, and Immigration: Prevention and Transcultural Support for Care within the Habitat Jeune Network of the Grand Est Region.

**Sponsor:**

University of Lorraine

34 Cours Léopold

BP 25233

54052 NANCY cedex

**Principal Investigator / Scientific Project Leader – Laboratory:**

Aziz Essadek

**Contact Persons for More Information:**

Aziz Essadek: [aziz.essadek@univ-lorraine.fr](mailto:aziz.essadek@univ-lorraine.fr)

Antoine Frigaux: [antoine.frigaux@univ-lorraine.fr](mailto:antoine.frigaux@univ-lorraine.fr)

Maha Najdini: [maha.najdini@univ-lorraine.fr](mailto:maha.najdini@univ-lorraine.fr)

**Nature and Objectives of the Project:**

This study aims to strengthen the effectiveness of mental health interventions.

**Methodology – Project description:**

This research seeks to deepen our understanding of perceptions and behaviors related to substance use among young people. A qualitative approach has been chosen through in-depth individual interviews. These interviews will allow us to explore young people's experiences and daily challenges, as well as their views on substance use and access to care.

**Study Process for Participants and Materials Used:**

The study consists of three individual interviews, each lasting approximately 60 minutes. The research process and consent form will be presented before the first interview. The first interview will be semi-structured. The second session will involve a projective test. The third will include a questionnaire and a summary of results. Audio recordings will be made using a dictaphone for transcription purposes. All recordings will be anonymized and destroyed after transcription.

**Consent and Study Participation:**

Participation is entirely voluntary. You may refuse to participate or withdraw at any time without consequence by notifying the principal investigator. You can also request that your collected data be destroyed.

You may ask questions at any time by contacting the scientific coordinator. If desired, you can be assisted by a trusted person throughout the study process.

**Possible Benefits and Risks:**

**Benefits:** Scientific, organizational, and public health contributions.
**Risks:** There are no significant risks, except for the time commitment. Some questions may address sensitive topics, but support will be available if needed. If urgent assistance is required, participants will be referred to appropriate medical and social services.

**Collected Data (Including Potential Audio and Image Recording):**

Types of Data Processed: Word-for-word transcriptions, voice recordings.

Sources of Data: Semi-structured interviews.

**Recipients of the Data:** Maha Najdini (Clinical Psychology PhD student). Antoine Frigaux (Clinical Psychology Researcher). Ariane Bazan (Thesis Supervisor). Aziz Essadek (Co-supervisor)

**Data Retention Period:**

**During the active research phase:** Three years

**After the research is completed:** Five years

**Confidentiality of Data:**

As part of this research, your personal data will be processed in accordance with the provisions of the General Data Protection Regulation No. 2016/679 (GDPR) and the amended Law 78-17, known as the “Informatics and Liberties” law. Your data will be anonymized. We will conceal your identity using a randomly assigned number. There will be no established correspondence between your name and this number. However, you have the right to object to your data being subject to automated processing. If you accept, you have the right to access your information in order to verify its accuracy and, if necessary, to correct, complete, or update it. You also have the right to object to its use, the right to data portability, the right to restriction of processing, and the right to the deletion of your data. To exercise these rights or to request the withdrawal of your consent, you can send an email to the following address: [aziz.essadek@univ-lorraine.fr](mailto:aziz.essadek@univ-lorraine.fr). This research is a study on human subjects aimed at developing biological and medical knowledge. It falls under Category 2 (interventional research with minimal risks and constraints). This research complies with the Reference Methodology MR-001of the French Data Protection Authority (CNIL) and its processing is registered with the University of Lorraine under the number 2024-A01534-43. This study is conducted in accordance with the provisions of the French Public Health Code relating to biomedical research. It has received favorable approval from the Committee for the Protection of Persons (CPP) under number 2024-A01534-43, dated November 18, 2024. All data and information concerning you will remain strictly confidential. They will only be accessible to the investigators of this research and to individuals appointed by the sponsor to monitor the quality of the study. If necessary, they may also be transmitted to authorized health authorities. In all cases, they will be used under conditions that ensure their confidentiality.

For any questions regarding data protection or complaints related to the processing of your personal data, you can contact the university's Data Protection Officer (DPO) at this address: dpo-contact@univ-lorraine.fr (Legal Affairs Department – Université de Lorraine, 34 Cours Léopold, BP 25233 - 54052 Nancy). Alternatively, you may contact the data protection authority in France, the Commission Nationale de l’Informatique et des Libertés (CNIL), at: <https://www.cnil.fr/fr/contacter-la-cnil-standard-et-permanences-telephoniques> or CNIL, 3 Place de Fontenoy, Service des plaintes, TSA 80715, 75334 PARIS CEDEX 7. In accordance with French law, the Sponsor (Université de Lorraine) has taken out Civil Liability Insurance covering the implementation of this study with MAIF (policyholder No. 4 676 033 J).

**Study Results Publication:**

Findings will be published in scientific articles, reports, and academic presentations. All data will be anonymized to ensure confidentiality.

**Appendix 2**

**Information notice (group intervention)**

**Project ATICC**

**N° ID-RCB : 2024-A01534-43**

Please read this notice carefully and ask any questions you may have. You can then decide whether or not you want to participate in this research.

**Title of the Study:** Addiction, Trauma, and Immigration: Prevention and Transcultural Support for Care within the Habitat Jeune Network of the Grand Est Region.

**Sponsor:**

University of Lorraine

34 Cours Léopold

BP 25233

54052 NANCY cedex

**Principal Investigator / Scientific Project Leader – Laboratory:**

Aziz Essadek

**Contact Persons for More Information:**

Aziz Essadek: [aziz.essadek@univ-lorraine.fr](mailto:aziz.essadek@univ-lorraine.fr)

Antoine Frigaux: [antoine.frigaux@univ-lorraine.fr](mailto:antoine.frigaux@univ-lorraine.fr)

Maha Najdini: [maha.najdini@univ-lorraine.fr](mailto:maha.najdini@univ-lorraine.fr)

**Nature and Objectives of the Project:**

We are launching a project to assess the beneficial impact of participation in the group program "Well-being coffee group" for young people in a specific context. The main objective is to improve the well-being and personal development of the participants.

**Methodology – Project description:**

This program aims to provide young people with a safe and supportive environment where they can share their experiences with confidence, acquire new skills, and develop effective strategies to cope with the challenges of daily life.

**Study Process for Participants and Materials Used:**

Participants will be invited to attend group sessions, with dates communicated in advance. Each session will last approximately an hour and a half and will take place at the residence facility starting from (to be specified later). A consent form will be provided to participants before each session. The collected data will include regular evaluations of the program’s impact on participants' well-being. The sessions will be led by external professionals and will cover a variety of topics related to different aspects of life. Each session will be interactive, encouraging idea exchange and mutual support among participants.

**Consent and Study Participation:**

Your participation in this study is entirely voluntary. You are free to refuse to participate or withdraw at any time by notifying the principal investigator. If you decide to withdraw, it will not affect your relationship with the institution or organization.

You may stop your participation in the study at any time, without justification and without any consequences, and you may request that your already collected data be deleted (withdrawal of your consent).

You may ask questions about the study at any time by contacting the scientific coordinator, within the constraints of the research plan.

**Possible Benefits and risks:**

**The potential benefits of this study include an improvement in the emotional and social well-being of the participants. No significant risks are associated with participation in this program, but support measures will be available if participants experience difficulties during the sessions.**

**Collected Data (Including Potential Audio and Image Recording):**

Categories of processed data: Observations, group content, scale results.

Sources: Written notes, voice recordings, standardized scales.

**Recipients of the Data:** Maha Najdini (Clinical Psychology PhD student). Antoine Frigaux (Clinical Psychology Researcher). Ariane Bazan (Thesis Supervisor). Aziz Essadek (Co-supervisor).

**Data Retention Period:**

**During the active research phase:** Three years

**After the research is completed:** Five years

**Confidentiality of Data:**

As part of this research, your personal data will be processed in accordance with the provisions of the General Data Protection Regulation No. 2016/679 (GDPR) and the amended Law 78-17, known as the “Informatics and Liberties” law. Your data will be anonymized. We will conceal your identity using a randomly assigned number. There will be no established correspondence between your name and this number. However, you have the right to object to your data being subject to automated processing. If you accept, you have the right to access your information in order to verify its accuracy and, if necessary, to correct, complete, or update it. You also have the right to object to its use, the right to data portability, the right to restriction of processing, and the right to the deletion of your data. To exercise these rights or to request the withdrawal of your consent, you can send an email to the following address: [aziz.essadek@univ-lorraine.fr](mailto:aziz.essadek@univ-lorraine.fr). This research is a study on human subjects aimed at developing biological and medical knowledge. It falls under Category 2 (interventional research with minimal risks and constraints). This research complies with the Reference Methodology MR-001of the French Data Protection Authority (CNIL) and its processing is registered with the University of Lorraine under the number 2024-A01534-43. This study is conducted in accordance with the provisions of the French Public Health Code relating to biomedical research. It has received favorable approval from the Committee for the Protection of Persons (CPP) under number 2024-A01534-43, dated November 18, 2024. All data and information concerning you will remain strictly confidential. They will only be accessible to the investigators of this research and to individuals appointed by the sponsor to monitor the quality of the study. If necessary, they may also be transmitted to authorized health authorities. In all cases, they will be used under conditions that ensure their confidentiality.

For any questions regarding data protection or complaints related to the processing of your personal data, you can contact the university's Data Protection Officer (DPO) at this address: dpo-contact@univ-lorraine.fr (Legal Affairs Department – Université de Lorraine, 34 Cours Léopold, BP 25233 - 54052 Nancy). Alternatively, you may contact the data protection authority in France, the Commission Nationale de l’Informatique et des Libertés (CNIL), at: <https://www.cnil.fr/fr/contacter-la-cnil-standard-et-permanences-telephoniques> or CNIL, 3 Place de Fontenoy, Service des plaintes, TSA 80715, 75334 PARIS CEDEX 7. In accordance with French law, the Sponsor (Université de Lorraine) has taken out Civil Liability Insurance covering the implementation of this study with MAIF (policyholder No. 4 676 033 J).

**Study Results Publication:**

Findings will be published in scientific articles, reports, and academic presentations. All data will be anonymized to ensure confidentiality. These results will contribute to expanding knowledge and informing public health practices and policies.

**Appendix 3**

**Consent Form**

**Project ATICC**

**N° ID-RCB : 2024-A01534-43**

Please read this form carefully and ask any questions you may find useful. You can then decide whether you want to participate in this study/research or not.

**Title of the Study:** Addiction, Trauma, and Immigration: Prevention and Transcultural Support for Care within the Habitat Jeune Network of the Grand Est Region.

**Sponsor:** University of Lorraine

**Adresse of sponsor**: 34 Cours Léopold, 54000 Nancy

I, the undersigned, ……………………………………………………………………….. (first and last name in capital letters), declare that I have understood the purpose and procedures of this study, which have been fully explained to me by ………………………………………………………………

□ I have received the specific information notice/letter, which I have had the opportunity to study carefully and which provides the contact details of the investigator to contact for any questions.

All my questions have been answered.

□ I have been given sufficient time for reflection before making my decision.

□ I agree to participate in this research under the conditions specified in the attached information letter. I remain free to withdraw from the study at any time without it affecting my relationship with the stakeholders of this study. I will inform the investigator if I choose to withdraw.

□ I have been informed that, in accordance with clinical study regulations, the Committee for the Protection of Persons 2024-A01534-43 has issued a favorable opinion for the conduct of this study on 18/11/2024.

□ I have also been informed that, in accordance with applicable law, an insurance contract (MAIF, policyholder No. 4 676 033 J) has been taken out by the research sponsor.

□ All data concerning me will remain confidential. I authorize access only to those involved in the research, those responsible for quality control on behalf of the sponsor, and representatives of health authorities.

□ I agree that the personal data necessary for the research may be collected during my participation in the study and processed in accordance with the provisions of the General Data Protection Regulation (GDPR No. 2016/679).

□ I have been clearly informed of the purpose of data processing (I have been explained how the data will be used) as well as the recipients of this data.

□ I acknowledge that, under the “Informatics and Liberties” Law of January 6, 1978, as amended, I have the right to access my personal data, as well as the right to rectify, oppose, transfer, and delete it. I can exercise these rights at any time by contacting the investigator.

□ I give my consent to participate in this research and to the processing of my personal data.

□ I may request additional information at any time from the persons responsible for the study, using the contact details provided in the information notice/letter.

The study requires an audio recording:

□ I authorize such recording to be carried out under the conditions specified in the information notice/letter.

□ I acknowledge that the audio recording will be destroyed upon transcription and that the anonymized interview data will be retained for three years.

My consent does not release the investigator and the sponsor from any of their responsibilities, and I retain all my rights guaranteed by law.

At the end of the research, I will be informed of the overall results of this study if I wish.

| To fill by the participant |
| --- |
| Date : ………………  Signature of the participant : |

| To fill by the investigator | |
| --- | --- |
| I, the undersigned, ………………………………………………………………………………. (name in capital letters), confirm that I have fully explained to the participant the purpose and procedures of this study, as well as its potential risks. I commit to ensuring compliance with the terms of this consent form, balancing respect for individual rights and freedoms with the requirements of scientific research.  Investigator’s phone number: …………………………………….. | |
| Signature of the investigator | Date : …………………………….. |

Made in two copies, one of which will be kept by the investigator and the other given to the participant.
